# Caractériser l'intersectionnalité au travail: des repères opérationnels pour soutenir les ergothérapeutes

**DOI:** 10.1177/00084174251383837

**Published:** 2025-10-21

**Authors:** Alexandra Lecours, Andrée-Anne Drolet, Lily Bellehumeur-Béchamp, Marie-Josée Drolet, Claude Vincent, Samuel Turcotte, Dimitri Léonard

**Keywords:** Analyse de concept, diversité identitaire, justice occupationnelle, participation occupationnelle, santé au travail, Concept Analysis, Diversity of Identity, Occupational Health, Occupational Justice, Occupational Participation

## Abstract

**Description.** Les ergothérapeutes accompagnent les personnes et les organisations vers une participation saine au travail. Dans ce contexte, il importe de considérer la multiplicité des identités, comme le genre, l’origine ethnique ou le handicap, dans l’analyse des inégalités et injustices occupationnelles que rencontrent les personnes au travail. Le concept d’intersectionnalité a le potentiel de soutenir les ergothérapeutes, mais il demeure difficile à reconnaitre dans la pratique. **But.** L’étude avait pour but d’identifier les caractéristiques opérationnelles du concept d’intersectionnalité au travail. **Méthodologie.** Selon un devis d’examen de la portée, 29 documents issus de disciplines variées ont été sélectionnés. Les données ont été extraites à l’aide d’une grille structurée et analysées selon une approche thématique. **Résultats.** Les résultats ont permis d’identifier cinq caractéristiques opérationnelles du concept, soit l’interrelation des identités, l’interaction entre désavantages et privilèges, les dynamiques de pouvoir, l’expérience subjective idiosyncratique ainsi que la relation à soi et aux autres. Nos résultats révèlent aussi que ces caractéristiques se doivent d’être considérées dans leur contexte, notamment historique. **Conclusion.** Cette étude offre un cadre aux ergothérapeutes pour analyser et intervenir dans des situations complexes, contribuant ainsi à promouvoir une participation au travail équitable et juste.

## Abstract (en)

**Description.** Occupational therapists support individuals and organizations in achieving healthy participation in the workplace. In this context, it is important to consider multiple identities, such as gender, ethnicity, or disability, when analyzing occupational inequalities and injustices that people encounter at work. The concept of intersectionality has the potential to support occupational therapists, but it remains difficult to recognize in practice. **Purpose.** The purpose of the study was to identify the operational characteristics of the concept of intersectionality at work. **Method.** Based on a scoping review design, 29 documents from various disciplines were selected. Data were extracted using a structured grid and analyzed using a thematic approach. **Results.** The results identified five operational characteristics of the concept: the interrelation of identities, the interaction between disadvantages and privileges, power dynamics, idiosyncratic subjective experience, and the relationship to oneself and others. Our results also reveal that these characteristics must be considered within their context, particularly their historical context. **Conclusion.** This study provides occupational therapists with a framework for analyzing and intervening in complex situations, thereby helping to promote equitable participation in the workplace.

## Introduction

Les ergothérapeutes sont qualifiés pour accompagner les personnes et les organisations dans la préservation d’une saine participation au travail, que ce soit en prévention des maladies et lésions professionnelles ou lors de la reprise du travail après une absence (Buchanan & van Niekerk, 2022; Drummond et al., 2020). Or, la diversification des profils identitaires des personnes en emploi qui s’accentue au Canada depuis la dernière décennie est à considérer dans la façon dont les ergothérapeutes agissent en regard de l’occupation du travail et des personnes qui y sont impliquées. Avec le vieillissement démographique, la mondialisation et l’intensification de l’immigration, la main-d’œuvre canadienne est de plus en plus diversifiée en termes d’âges et d’origines ethniques (Tamunomiebi & John-Eke, 2020). De plus, l’avènement des politiques d’équité, de diversité et d’inclusion en emploi incite les milieux de travail à assurer une diversité en termes de sexes, de genres, de capacités, de handicaps ou d’orientation sexuelle parmi leur main-d’œuvre. Toutefois, la littérature fait état de formes de discrimination en milieu de travail qui maintiennent la sous-représentation des groupes marginalisés malgré la diversité apparente (Smith et al., 2012). La question de la diversité en emploi demeure ainsi complexe.

La diversité des identités peut procurer des avantages pour la participation à l’occupation du travail. Par exemple, Cho et Mor Barak (2008) ont démontré qu’une équipe de travail empreinte de diversité permet à une plus grande variété d’individus de s’identifier à leurs collègues de travail, ce qui augmente leur sentiment d’appartenance et leur engagement au travail. En outre, les milieux de travail caractérisés par la diversité tendent à innover, en raison de la créativité de leurs équipes et d’une culture organisationnelle flexible (Downie, 2010), des facteurs susceptibles d’influencer positivement l’expérience de travail des personnes.

Toutefois, la diversité peut aussi entrainer des défis pour les milieux de travail. Au quotidien, la diversité des pensées, de raisonnements, de valeurs et de modes opératoires peuvent mener à des enjeux de communication, des conflits, des tensions ou encore à des incidents et accidents, ce qui risque de miner la qualité de la participation au travail (Bah, 2015). La diversité des identités en milieu de travail est ainsi susceptible de générer de la discrimination (Bah, 2015) et d’affecter la santé, le bien-être (Okechukwu et al., 2014) ou la participation occupationnelle (Trentham & Neysmith, 2018) des personnes. Qui plus est, des auteurs en ergothérapie reconnaissent que l’intersection entre les caractéristiques identitaires minoritaires des personnes est susceptible d’être à l’origine de préjudices subis par ces dernières, risquant de leur faire vivre des injustices occupationnelles (Pooley & Beagan, 2021; Restall, 2024). Quand une personne présente plusieurs identités minoritaires (p. ex., être une femme et appartenir à une minorité ethnique ou être un individu issu de l’immigration et être âgé), son expérience devient plus complexe et les risques de discrimination augmentent (Remedios & Snyder, 2018), notamment dans le domaine du travail (Côté et al., 2020). Il est ainsi nécessaire d’étudier les caractéristiques identitaires de manière intégrée, plutôt que de façon isolée pour comprendre pleinement le portrait des inégalités et injustices occupationnelles (Mujtaba, 2007).

À cet égard, le concept d’intersectionnalité a été initialement proposé dans le domaine du droit aux États-Unis par Crenshaw (1989) afin d’expliquer l’expérience multidimensionnelle des femmes noires confrontées simultanément au racisme et au sexisme. Selon ces travaux, une analyse non intégrée de ces deux aspects déforme la réalité vécue par ces femmes et renforce davantage leur marginalisation sociale (Crenshaw, 1989). À travers l’étude de cas juridiques impliquant le travail des femmes noires dans les domaines du transport et de la fabrication, Crenshaw met en relief que l’oppression vécue par ces personnes ne peut être réduite à une simple addition de discriminations raciale et sexiste dans l’analyse des relations de travail. Il faut comprendre comment ces discriminations s’entrelacent pour créer une marginalisation unique. Selon cette autrice phare du concept, l’intersectionnalité se définit comme un cadre analytique permettant de comprendre comment les différentes formes de discrimination se croisent et créent des expériences uniques d’oppression (Crenshaw, 1991). Avec les années, des travaux ont permis d’élargir le concept d’intersectionnalité à d’autres caractéristiques identitaires comme la religion et l’orientation sexuelle (Rodriguez et al., 2013) ou encore la race et les incapacités (Mereish, 2012). L’évolution et l’intérêt pour le concept depuis la fin des années 1980 a fait en sorte qu’il est étudié et mobilisé dans divers contextes, comme en sociologie pour l’étude des politiques sociales (Davis, 2008), en éducation pour comprendre et réduire la discrimination dans les écoles (Bešić, 2020) ou en santé publique pour mieux comprendre l’accès aux soins pour différentes populations (Ogungbe et al., 2019). Ces travaux ont permis de formuler des définitions contemporaines du concept, la plupart basées sur les travaux originaux de Crenshaw. Par exemple, Collins and Bilge (2020), dans le champ de la sociologie, définissent l’intersectionnalité comme la manière dont les rapports de pouvoir qui se croisent influencent les relations sociales au sein des sociétés ainsi que les expériences humaines dans la vie quotidienne. En psychologie, Warner (2008) définit le concept comme l’idée selon laquelle les identités sociales interagissent pour former des significations et des expériences qualitativement distinctes.

### Intersectionnalité et ergothérapie

Bien que le concept d’intersectionnalité ait été initialement développé dans des disciplines connexes, plusieurs auteurs en ergothérapie et en science de l’occupation s’en sont progressivement saisis au fil des années. Par exemple, un nombre croissant de travaux invitent les ergothérapeutes à reconnaître et à nommer les causes structurelles des inégalités, à s’engager activement en faveur de la justice occupationnelle, et à demeurer attentifs aux dynamiques d’exclusion susceptibles d’entraver la participation occupationnelle des personnes, notamment au travail (p. ex., Angell, 2014; Farias et al., 2016; Gupta & Garber, 2017). Certains auteurs vont encore plus loin, affirmant que le cadre collaboratif de la justice occupationnelle participative met en évidence la nécessité de reconnaître et de corriger les inégalités structurelles qui limitent la participation occupationnelle (Whiteford et al., 2017). Des écrits ont également été publiés sur des concepts connexes, comme l’oppression (Pooley & Beagan, 2021), le sanisme (LeBlanc & Kinsella, 2016) ou le capacitisme (Yao et al., 2022), contribuant à mettre en lumière les mécanismes de discrimination liés aux identités sociales et aux rapports de pouvoir.

Par ailleurs, certains travaux ont directement porté sur le concept d’intersectionnalité dans une perspective ergothérapique (p. ex., Angell, 2014; Gerlach, 2015; Nirmul et al., 2023; Smith et al., 2025). Par exemple, Smith et collaborateurs (2025) ont mené une étude visant à informer comment le concept d’intersectionnalité peut guider l’enseignement, la pratique et la recherche en ergothérapie. Les résultats de leur examen de la portée invitent les ergothérapeutes à adopter une posture critique face aux inégalités structurelles, à reconnaître les dynamiques d’oppression dans leurs pratiques, à promouvoir la justice et à réfléchir aux manières dont ces oppressions peuvent être reproduites, même de façon involontaire, dans leurs interactions professionnelles. Dans le même sens, les travaux de Gerlach (2015) soulignent la pertinence d’une approche intersectionnelle pour soutenir la réflexion critique sur la pratique auprès de populations marginalisées. Cette approche permet une analyse fine et contextualisée des situations occupationnelles complexes vécues par les personnes. L’intersectionnalité offre ainsi un cadre essentiel pour reconnaître les expériences plurielles et multidimensionnelles des individus marginalisés, et adapter les interventions ergothérapiques pour favoriser une participation occupationnelle équitable et inclusive, notamment dans les milieux de travail.

### Critiques du concept et pertinence de l’étude

Malgré la reconnaissance scientifique de la pertinence du concept d’intersectionnalité, plusieurs critiques ont été soulevées au fil des années. L’une des principales concerne sa polysémie conceptuelle : l’intersectionnalité est à la fois définie comme une théorie, un cadre analytique, un paradigme, une stratégie de revendication, une métaphore ou un outil heuristique (Davis, 2008). Cette multiplicité d’interprétations contribue à une confusion conceptuelle et à une variabilité dans les usages qui en sont faits en recherche (Davis, 2008). De plus, la complexité inhérente au concept rend son opérationnalisation difficile, ce qui complique la reconnaissance concrète des phénomènes qu’il permet de mettre en lumière (Carastathis, 2008; Nash, 2017). À ce sujet, des auteurs ont noté la difficulté pour les ergothérapeutes de mobiliser un concept théorique en pratique, notamment parce qu’ils sont souvent abstraits (Ikiugu et al., 2009; O'Neal et al., 2007). Pour y remédier, une recommandation a été émise afin de rendre les concepts plus accessibles, notamment en identifiant leurs caractéristiques opérationnelles (Nash, 2017). Les caractéristiques opérationnelles permettent d’identifier un concept dans la réalité et de le distinguer de concepts apparentés. Ainsi, la reconnaissance de ces caractéristiques permet d’opérationnaliser un concept en identifiant le phénomène qu’il désigne dans la réalité (Walker & Avant, 2019). Appliquée au concept d’intersectionnalité et à l’ergothérapie, cette approche rejoint les recommandations visant à mieux outiller les ergothérapeutes pour repérer les situations d’oppression vécues par les clientèles qu’ils accompagnent. Elle s’inscrit également dans la promotion de la compétence « Culture, équité et justice » du Référentiel de compétences pour les ergothérapeutes au Canada (ACORE, 2021), qui vise à renforcer la capacité des ergothérapeutes à intervenir de manière équitable et culturellement sécuritaire.

La nécessité de contextualiser le concept d’intersectionnalité et d’adopter une approche tenant compte des variations propres à chaque contexte est reconnue comme essentielle pour en assurer une mobilisation pratique plus pertinente et efficace (Carastathis, 2008). Cette exigence est d’autant plus importante que le concept, issu des travaux fondateurs de Crenshaw (1989), trouve une résonance particulière dans les milieux de travail, où les dynamiques d’oppression et de discrimination peuvent émerger à l’intersection de multiples identités (Acker, 2011; Rodriguez et al., 2016). C’est dans cette optique que notre étude se concentre sur l’intersectionnalité dans le contexte du travail. Examiner la diversité des identités et la manière dont elles coexistent au sein des environnements professionnels apparaît fondamental pour soutenir adéquatement les personnes et les organisations (Mujtaba, 2007), une mission au cœur de la pratique des ergothérapeutes. Ainsi, notre étude avait pour but d’identifier les caractéristiques opérationnelles du concept de l’intersectionnalité au travail.

## Méthodologie

### Devis et posture de l’équipe de recherche

Afin de répondre à notre objectif de recherche, nous avons effectué un examen de la portée pour obtenir une vision d’ensemble de la littérature scientifique disponible concernant le concept de l’intersectionnalité dans le domaine du travail, en évaluant sa quantité, sa portée et sa nature (Peters et al., 2020). En effet, ce devis est reconnu pour sa capacité à identifier les caractéristiques-clés d’un concept à l’intérieur d’un contexte particulier (Munn et al., 2022), ce qui est parfaitement aligné avec notre objectif. Pour accroître la rigueur scientifique de cet article, le « *Preferred Reporting Items for Systematic Reviews and Meta-Analyses Extension for Scoping Reviews (PRISMA-ScR) checklist* » a été utilisé pour rapporter les informations (Tricco et al., 2018).

Étant donné que notre étude porte sur un sujet lié aux caractéristiques identitaires et que l’examen de la portée repose en partie sur l’interprétation des écrits scientifiques, il est essentiel de prendre en compte la composition de notre équipe, puisque celle-ci peut influencer les résultats que nous proposons (Collins, 2015). Nous sommes une équipe composée de sept personnes, de couleur blanche, provenant de l’Amérique du Nord et de l’Europe et ayant poursuivi des études universitaires. Cette homogénéité sur le plan ethnoracial et éducatif peut façonner nos perspectives, notamment la manière dont nous analysons les concepts et la sensibilité que nous avons envers certaines réalités vécues par des populations marginalisées. Toutefois, notre équipe présente une diversité concernant l’âge, le genre, l’orientation sexuelle et la culture, ce qui permet d’intégrer une pluralité de points de vue et d’expériences dans notre analyse. De plus, nous provenons de domaines de spécialité variés, soit l’ergothérapie, l’administration, la philosophie, la psychologie et la sociologie, ce qui nous permet d’aborder notre objet d’étude sous différents angles. Cette complémentarité disciplinaire favorise une compréhension holistique du phénomène étudié et enrichit notre interprétation des résultats. Nous reconnaissons néanmoins que nos perspectives sont influencées par nos trajectoires personnelles et académiques, et nous nous engageons à adopter une posture réflexive afin de minimiser les biais pouvant découler de notre positionnalité.

### Procédure

L’examen de la portée a été mené en cinq étapes.

#### Étape 1 : Identification de la question de recherche

Notre question de recherche « Quelles caractéristiques permettent de reconnaître l’intersectionnalité dans les situations de travail» se justifie du fait que la question de recherche doit être large, mais aussi inclure des spécificités concernant le but de l’étude et de son contexte (Peters et al., 2020).

#### Étape 2 : Recherche documentaire

La stratégie de recherche documentaire a été développée par l’équipe de recherche en collaboration avec une bibliothécaire pour s’assurer de la précision et de la rigueur de la démarche (Daudt et al., 2013). Premièrement, des combinaisons de mots-clés liés à l’intersectionnalité et au travail comme « *intersectionality* » et « *workplace* » ont été utilisées. Puis, en raison du caractère interdisciplinaire du concept, des bases de données spécialisées dans les domaines de la santé et de la réadaptation (Academic Search Complete (EBSCO), CINHAL Complete (EBSCO), Scopus), de la sociologie (Sociological Abstract et SocIndex), de la psychologie (APA PsycINFO) et de l’administration (ABI/INFORM GLOBAL) ont été ciblées. Deuxièmement, une recherche secondaire a été effectuée en examinant manuellement les listes de références des documents sélectionnés afin d'identifier d'autres documents pertinents. Troisièmement, une recherche manuelle a été effectuée dans Google en utilisant les mots-clés de la stratégie de recherche. Les titres et les brèves descriptions trouvés dans les dix premières pages ont été examinés (Godin et al., 2015). Cette stratégie de recherche documentaire en plusieurs étapes permet d’assurer une couverture exhaustive de la littérature disponible. Cette étape de recherche documentaire a été réalisée par deux personnes de l’équipe de recherche et validée par l’équipe entière.

#### Étape 3 : Sélection des documents

**Figure 1.**
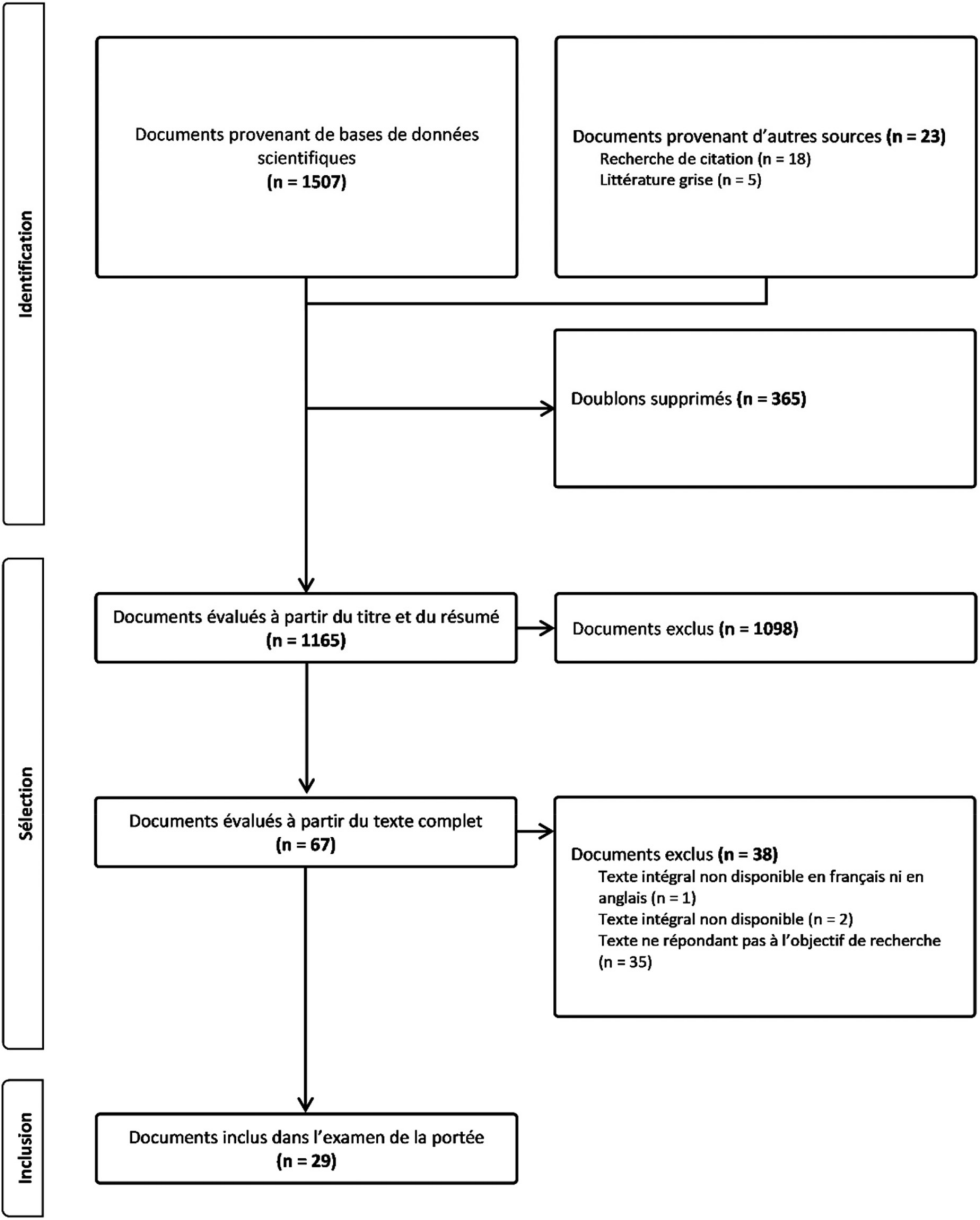
Logigramme du processus de sélection des documents.

Les documents identifiés ont été intégrés dans un logiciel de gestion des références Endnote, puis importés dans Covidence, une plateforme web dédiée à la gestion des études de type recension d’écrits. Après l’élimination des doublons, la pertinence des documents a été déterminée sur la base de la lecture du titre, du résumé et des mots-clés. Pour ce faire, les critères d’inclusion suivants ont été utilisés : (1) le document porte sur l’intersectionnalité ; (2) le document s’inscrit dans le contexte du travail ; (3) le document est publié en 2000 ou après. En accord avec les recommandations pour la conduite d’examens de la portée, tous les types de documents (scientifiques, professionnels, gouvernementaux, grand public) ont été inclus dans l’étude (Peters et al., 2020). Les critères d’exclusion suivants ont également guidé la section des documents : (1) le document en version complète n’est pas disponible ; (2) le document n’est publié ni en anglais ni en français. Ces critères ont été prétestés pour la sélection de vingt documents pour en assurer la clarté. Dans un deuxième temps, les documents retenus ont été lus en entier pour assurer leur pertinence en regard de l’objectif de l’étude. Ce processus de sélection des documents a été accompli par deux membres de l’équipe de recherche ; un troisième membre était sollicité en cas de désaccord ou de doute. Des réunions de concertation entre les membres de l’équipe de recherche ont renforcé leur réflexivité et contribué à réduire le risque de biais personnels (Padgett, 2017). Suivant ce processus de recherche systématique, 29 documents ont été inclus dans l’analyse, comme présentés à la figure 1.

#### Étape 4 : Extraction des données

L’extraction des informations contenues dans les documents sélectionnés a été réalisée à l’aide d’une grille incluant des informations descriptives à propos des documents (p. ex., auteurs, pays), des informations méthodologiques (p. ex., devis, participants) et des résultats (p. ex., caractéristiques définissant le concept). La grille a été prétestée par deux membres de l’équipe de recherche, de façon indépendante, lors de l’extraction des cinq premiers documents sélectionnés. Une rencontre de concertation entre les deux membres a eu lieu après l’extraction de chacun de ces documents pour mettre en commun l’information extraite et pour ajuster la grille. Après ce pré-test, les deux membres ont établi un consensus sur la structure de la grille d’extraction. Cette version finale a été utilisée pour extraire les informations contenues dans les vingt-quatre autres manuscrits, où l’information a été extraite par un seul membre de l’équipe. Toutefois, des rencontres régulières entre les membres de l’équipe ont eu lieu pour assurer la rigueur dans le processus d’extraction.

#### Étape 5 : Analyse des résultats

Des analyses statistiques descriptives ont été réalisées pour dénombrer les informations de nature descriptive (p. ex., nombre et types de documents sélectionnés). Ensuite, une analyse de contenu a été menée pour catégoriser les informations recensées et identifier les caractéristiques opérationnelles du concept d’intersectionnalité au travail. Cette approche analytique est recommandée pour les examens de la portée visant à identifier les caractéristiques définissant un concept (Pollock et al., 2023). Dans un premier temps, une lecture entière du corpus (c.-à-d., données extraites via les grilles d’extraction) a été réalisée. Plusieurs autres lectures ont été ensuite menées pour assurer un sentiment d’immersion des membres de l’équipe de recherche dans le corpus de données. Selon une approche inductive, le codage initial a permis d’attribuer des codes descriptifs aux idées contenues dans les données. Ensuite, ces codes étaient regroupés en catégories. Plusieurs cycles d’application des données de la grille d’extraction à la structure générale proposée ont permis d’affiner le processus d’analyse et de formuler les caractéristiques opérationnelles du concept d’intersectionnalité au travail. En accord avec le devis d’examen de la portée, tous les documents ont été analysés en leur accordant la même importance. Aucune distinction n’a été faite selon le type de document et aucune évaluation de la qualité des études n'a été faite (Arksey & O'Malley, 2005). Cette étape a été réalisée par deux membres de l’équipe de recherche et des rencontres avec toute l’équipe ont eu lieu pour assurer la rigueur du processus.

### Éthique

L’approbation éthique par un comité n’est pas requise pour cette étude, considérant qu’il s’agit de l’analyse de données tirées de documents déjà publiés.

## Résultats

La présente section expose les résultats de l’étude. Une description des documents sélectionnés est d’abord présentée, suivie d’une synthèse narrative des caractéristiques opérationnelles du concept d’intersectionnalité au travail. Enfin, des éléments sur les contextes sociétal et historique qui influencent la manifestation de l’intersectionnalité au travail sont abordés.

### Description des documents

Le processus de recherche documentaire a permis d’inclure 29 documents. Parmi les documents sélectionnés, 76% ont été publiés dans les 10 dernières années (n = 22), et majoritairement en anglais (n = 23). Plus de la moitié sont des articles scientifiques (n = 21) et 48% sont issus du domaine de l’administration. Les populations étudiées dans les documents proviennent de plusieurs pays, dont les États-Unis (n = 7) et le Canada (n = 4). Finalement, chaque texte abordait plusieurs diversités et contextes de travail. Le Tableau 1 décrit les caractéristiques des documents sélectionnés et l’Annexe 1 présente les 29 références consultées.

**Tableau 1.** Caractéristiques des documents sélectionnés

Auteurs, année	Champs de littérature/ discipline	Pays	Type de document	Contexte de l’étude	Caractéristiques identitaires	Caractéristiques opérationnelles du concept
Acciari (2020)	Sociologie	Brésil	Article scientifique	L’expérience des travailleuses domestiques du Brésil	- Genre - Ethnicité - Statut socio-économique	- Interrelation entre les identités - Relation à soi et aux autres
Acciari (2021)	Sociologie	Brésil	Article scientifique	L’expérience des travailleuses domestiques du Brésil	- Genre - Ethnicité Statut socio-économique	- Interrelation entre les identités - Relation à soi et aux autres - L’expérience subjective idiosyncratique
Acker (2011)	Sociologie	N/S^2^	Chapitre de livre	N/S	- N/S	- Interrelation entre les identités - Interaction entre désavantages et privilèges​ - Relation à soi et aux autres
Alberti and Iannuzzi (2020)	Gestion/ administration	Italie	Article scientifique	L’expérience des travailleurs de l’hôtellerie en Italie	- Genre - Ethnicité - Statut d’immigrant	- Interrelation entre les identités - Dynamiques de pouvoir - Interaction entre désavantages et privilèges​ - Relation à soi et aux autres
Alt et al. (2024)	Psychologie	États-Unis	Article scientifique	L’expérience des travailleuses d’origine asiatico-américaines	- Genre - Ethnicité	- Interrelation entre les identités - Dynamiques de pouvoir ​ - Interaction entre désavantages et privilèges​​
Arifeen and Gatrell (2013)	Gestion/ administration	Grande-Bretagne	Article scientifique	L’expérience des travailleuses britanniques d’origine pakistanaise	- Genre - Ethnicité - Religion - Nationalité	- Interrelation entre les identités - Dynamiques de pouvoir - L’expérience subjective idiosyncratique
Borland and Bruening (2010)	Gestion/ administration	États-Unis	Article scientifique	L’expérience des femmes noires entraîneuses-cheffes dans le milieu du basketball	- Genre - Ethnicité - Orientation sexuelle	- Interrelation entre les identités ​ - L’expérience subjective idiosyncratique - Relation à soi et aux autres
Browne and Misra (2003)	Sociologie	États-Unis	Article scientifique	N/S	- Genre - Ethnicité	- Dynamiques de pouvoir - Interaction entre désavantages et privilèges - L’expérience subjective idiosyncratique
Callico (2020)	Gestion/ administration	N/S	Site web	N/S	- N/S	- Interrelation entre les identités - L’expérience subjective idiosyncratique - Dynamiques de pouvoir
Chilakala et al. (2022)	Santé	N/S	Article scientifique	L’expérience des femmes noires exerçant la médecine en contexte hospitalier	- Genre - Ethnicité	- Interrelation entre les identités - Dynamiques de pouvoir ​ - L’expérience subjective idiosyncratique​
Corlett and Mavin (2014)	Gestion/ administration	N/S	Article scientifique	N/S	- N/S	- Interrelation entre les identités - Dynamiques de pouvoir - L’expérience subjective idiosyncratique ​
De los Reyes (2017)	Économie	N/S	Article scientifique	N/S	- N/S	- Interrelation entre les identités ​ - Dynamiques de pouvoir ​ - Interaction entre désavantages et privilèges​​
Dhanani et al. (2024)	Gestion/ administration	N/S	Article scientifique	L’expérience des travailleur·ses blanc·hes et noir·es issu·es des minorités sexuelles	- Ethnicité - Orientation sexuelle	- Interrelation entre les identités - Relation à soi et aux autres
DiStasio (s.d.)	Gestion/ administration	Canada	Site web	N/S	- N/S	- Interrelation entre les identités - L’expérience subjective idiosyncratique
Fuentes et al. (2024)	Santé et réadaptation	N/S	Article scientifique	L’expérience des personnes racisées en situation de handicap en milieu de travail	- Ethnicité - Capacité	- Interrelation entre les identités - L’expérience subjective idiosyncratique​
Gallot et al. (2020)	Sociologie	N/S	Article scientifique	N/S	- N/S	- Dynamiques de pouvoir
Institute for Gender and the Economy (2019)	Économie	Canada	Site web	N/S	- N/S	- Interrelation entre les identités - Dynamiques de pouvoir
Kriger et al. (2021)	Gestion/ administration	Canada	Site web	Recommandations pour les praticien·nes du sport	- N/S	- Interrelation entre les identités ​
Lavaysse et al. (2018)	Santé publique	États-Unis	Article scientifique	N/S	- N/S	- Interrelation entre les identités - L’expérience subjective idiosyncratique ​​
Luiz and Terziev (2024)	Gestion/ administration	Afrique du sud	Article scientifique	L’expérience de gestionnaires de niveau intermédiaire LGBT en Afrique du Sud	- Genre - Ethnicité - Orientation sexuelle	- Interrelation entre les identités ​ - Dynamiques de pouvoir - Interaction entre désavantages et privilèges​ - L’expérience subjective idiosyncratique ​​
McDowell and Carter-Francique (2017)	Gestion/ administration	États-Unis	Article scientifique	L’expérience des directeurs athlétiques aux États-Unis	- Genre - Ethnicité	- Interrelation entre les identités
Mooney et al. (2017)	Gestion/ administration	Nouvelle-Zélande	Article scientifique	L’expérience des employé·es du secteur de l’hôtellerie	- Genre - Ethnicité - Âge	- Interaction entre désavantages et privilèges​ - L’expérience subjective idiosyncratique​
Randstad (2020)	Gestion/ administration	Canada	Site web	N/S	- N/S	- Interrelation entre les identités ​ - L’expérience subjective idiosyncratique ​​
Rodriguez et al. (2016)	Gestion/ administration	N/S	Article scientifique	L’expérience des employé·es dans des milieux de travail influencés par l’économie néolibérale et le féminisme corporatif	- N/S	- Interrelation entre les identités - Dynamiques de pouvoir - Interaction entre désavantages et privilèges​ - L’expérience subjective idiosyncratique
Rosette et al. (2019)	Comportement organisationnel	États-Unis	Article scientifique	N/S	- Genre - Ethnicité	- Interrelation entre les identités - Dynamiques de pouvoir ​ - L’expérience subjective idiosyncratique ​​
Salter et al. (2021)	Psychologie	N/S	Article scientifique	N/S	- Genre - Ethnicité - Orientation sexuelle - Âge	- Interrelation entre les identités
Summerville (2022)	Philosophie en sciences organisationnelles	États-Unis	Thèse	N/S	- Genre - Ethnicité	- Interrelation entre les identités ​ - L’expérience subjective idiosyncratique
Van Buren III (2015)	Gestion/ administration	N/S	Chapitre de livre	N/S	- N/S	- Interrelation entre les identités ​ - Dynamiques de pouvoir ​ - L’expérience subjective idiosyncratique - Relation à soi et aux autres
Wang (2015)	Sociologie	Chine	Article scientifique	L’expérience des femmes migrantes au stade de l’embauche dans le marché du travail urbain	- Genre - Statut d’immigrant	- Interrelation entre les identités - Dynamiques de pouvoir - L’expérience subjective idiosyncratique - Relation à soi et aux autres

### Caractéristiques opérationnelles du concept de l’intersectionnalité au travail

L’analyse des données extraites des 29 documents sélectionnés a permis de faire émerger 5 caractéristiques : (1) Interrelation des identités, (2) Interaction entre désavantages et privilèges, (3) Dynamiques de pouvoir, (4) Expérience subjective idiosyncratique, et (5) Relation à soi et aux autres.

#### Interrelation entre les identités

L’un des fondements du concept d’intersectionnalité réside dans l’interrelation entre les différentes identités ou caractéristiques identitaires. Cette perspective met en lumière la manière dont des identités multiples, comme le genre, le sexe, la race, l’ethnicité, l’orientation sexuelle ou le handicap, s’entrecroisent pour former des systèmes interconnectés (Acciari, 2021; Browne & Misra, 2003). Ainsi, des identités souvent traitées séparément dans les discours sociaux ou administratifs sont, en réalité, vécues de manière imbriquée. Par exemple, Chilakala et collaborateurs (2022) illustrent cette idée en affirmant que « […] la race et le sexe, bien que souvent séparés sur le papier, sont étroitement liés en réalité, illustrant ainsi la notion d'intersectionnalité »^1^ (p. 109). Cette interaction entre les identités signifie qu’elles ne s’expriment pas isolément, mais s’influencent mutuellement dans le vécu des individus (Fuentes et al., 2024). Elles peuvent ainsi se renforcer, s’entrelacer ou se chevaucher selon les contextes (Fuentes et al., 2024; Salter et al., 2021). C’est précisément cette dynamique relationnelle, plutôt que les identités prises individuellement, qui façonne l’expérience des personnes (Acciari, 2020; Fuentes et al., 2024). Dans cette optique, Acciari (2020) souligne que « ces témoignages [des travailleuses] montrent la forte interconnexion des rapports sociaux de genre, race et classe dans le travail […]. La combinaison particulière de ces trois vecteurs d’oppression a conduit à la création d’une sous-classe de travailleuses, dévalorisées et précarisées » (p. 128). La perspective interactionnelle de l’intersectionnalité propose un cadre utile pour analyser la manière dont les formes d’oppression peuvent se manifester simultanément ou de manière séquentielle (Borland & Bruening, 2010). Par ailleurs, certaines caractéristiques identitaires sont invisibles, comme la religion ou l’orientation sexuelle, ce qui peut rendre plus difficile leur reconnaissance et la prise en compte des inégalités qui y sont associées dans les milieux de travail. Comme le soulignent Dhanani et collaborateurs (2024) « les inégalités invisibles au travail devraient être élargies pour incorporer l'intersection de multiples identités stigmatisées comme une forme d'inégalité qui a été jusqu'à présent négligée » (p. 23).

#### Interaction entre désavantages et privilèges

L’intersectionnalité prend également en compte la manière dont les rapports de privilèges et de désavantages s’entrelacent dans les contextes de travail. Ces rapports sont souvent issus de constructions sociales qui valorisent ou dévalorisent certaines identités, parfois de façon implicite, parfois explicite (Browne & Misra, 2003). Plutôt que de se présenter de manière distincte, ces rapports s’entrecroisent, produisant des effets différenciés selon les combinaisons identitaires des individus (Luiz & Terziev, 2024). Ce chevauchement peut entraîner des avantages pour certaines personnes, tout en créant des désavantages pour d’autres (Rodriguez et al., 2016). Cela peut s’illustrer de diverses façons comme le suggèrent Browne and Misra (2003)

Les expériences des femmes d’origine latine sur le marché du travail sont liées aux expériences des femmes blanches. Ainsi, les femmes blanches sont plus susceptibles d’être considérées comme des travailleuses professionnelles que les Latinas, et ce sont les femmes blanches qui bénéficient de ce privilège (p.490–491).

Dans ce scénario, un groupe de travailleuses bénéficient d’un privilège qui se traduit néanmoins comme un désavantage pour un autre groupe. Ces notions de désavantages et de privilèges interagissent et s’influencent mutuellement (Browne & Misra, 2003; Rodriguez et al., 2016). Il importe également de noter qu’un même individu peut être simultanément porteur de privilèges et de désavantages. Comme mentionné par Luiz and Terziev (2024) :

[certaines personnes] présentent simultanément des avantages (avoir la peau de couleur blanche) et des désavantages (leur homosexualité). Selon les contextes du lieu de travail, elles pourraient présenter l'un ou l'autre de ces éléments, à savoir un avantage ou un désavantage, et pourraient passer de l'état d'oppresseur blanc à celui d'opprimé homosexuel dans la même journée. Cela résulte en un sentiment d’aliénation, car elles vivent le statut d’oppresseur, tout en subissant simultanément une discrimination fondée sur leur sexualité (p.308).

Enfin, les personnes qui bénéficient principalement de privilèges peuvent avoir plus de difficulté à percevoir les désavantages vécus par d’autres (Acker, 2011; Van Buren III, 2015). Une perspective intersectionnelle permet de développer cette prise de conscience et de tendre vers des relations de travail plus équitables (Van Buren III, 2015).

#### Dynamiques de pouvoir

L’intersectionnalité met également en lumière les dynamiques de pouvoir dans les milieux de travail. Ces dynamiques s’expriment à travers des interactions inégales entre individus ou groupes, et influencent la répartition des ressources, des responsabilités ou des opportunités. Cette caractéristique concerne notamment les structures sociales et les hiérarchies. Elles révèlent comment certaines personnes, en raison de leur statut ou de leur autorité, peuvent exercer un pouvoir sur d’autres. En effet, « l'intersectionnalité est avant tout une question de pouvoir : qui l'a et qui n'en a pas. L’intersectionnalité se concentre sur la manière dont les différences humaines se croisent de manière à provoquer l’assujettissement » (Van Buren III, 2015, p.360). Cette perspective souligne que les normes organisationnelles sont souvent façonnées par des groupes dominants qui définissent quelles identités sont valorisées et, à l’inverse, lesquelles sont perçues comme marginales. Comme l’explique De los Reyes (2017), « les pratiques qui positionnent les personnes dans des catégories de classe, de sexe, d'origine ethnique et d'âge différenciées expriment non seulement le pouvoir de définir qui représente la main-d'œuvre normale, acceptée et attrayante, mais aussi qui est considéré comme déviant, subordonné et indésirable » (p. 16). Ce sont les dynamiques sociales qui attribuent ou retirent de l’autorité, du contrôle ou de la reconnaissance, souvent selon des critères structuraux et selon les contextes. Par exemple, l’article d’Alberti et Iannuzzi (2020) suggère que « puisque le travail hôtelier est fondamentalement une activité orientée vers la clientèle, non seulement le contexte organisationnel, mais aussi les stratégies et les présupposés des employeurs à l’égard des préférences des clients influencent de manière déterminante les attentes quant aux identités des travailleurs pour certains emplois » (p. 1168). Ces dynamiques de pouvoir ont des conséquences concrètes sur l’inclusion, notamment dans les processus de recrutement, de promotion ou de reconnaissance professionnelle (Acker, 2011). Ainsi, elles contribuent à reproduire les inégalités structurelles en place dans les milieux de travail, même en l’absence d’intention explicite de discrimination.

#### Expérience subjective idiosyncratique

L’expérience subjective idiosyncratique désigne la manière personnelle et contextualisée dont chaque individu perçoit, vit et interprète les effets de ses identités multiples au sein de son environnement professionnel (Wang, 2015). Cette expérience est marquée par son unicité : chaque personne traverse une réalité distincte façonnée par une combinaison singulière de caractéristiques identitaires (Chilakala et al., 2022), lesquelles interagissent avec des dynamiques sociales et professionnelles propres au contexte (Luiz & Terziev, 2024). Dans cette optique, les initiatives visant à contrer les inégalités doivent tenir compte de la diversité de vécus des personnes, comme le souligne Randstad (2020), pour qui « leurs expériences très différentes ne peuvent pas être abordées de la même manière et des solutions mieux adaptées sont nécessaires pour traiter la discrimination subie par chacune d’entre elles » (s. p.). La reconnaissance de cette diversité d’expériences est essentielle, car, comme le note Van Buren III (2015) « l’expérience humaine ne peut pas être facilement catégorisée sur la base d'un seul attribut ou caractéristique personnelle » (p.358). Autrement dit, l’expérience individuelle ne peut être comprise qu’en prenant en compte la complexité des identités en jeu et le contexte dans lequel elles s’expriment.

De plus, l’expérience subjective idiosyncratique permet de révéler l’impact différencié et souvent invisible que peut avoir l’intersection de plusieurs identités sur la santé au travail (Lavaysse et al., 2018). Malgré cette singularité, l’interprétation des écrits permet de suggérer certaines régularités parmi le vécu des personnes. Par exemple, certaines personnes peuvent vivre un sentiment d’isolement, ou percevoir que leurs compétences sont présumées insuffisantes ou excessives selon les stéréotypes associés à leurs identités (Chilakala et al., 2022). Par exemple, dans une étude portant sur les femmes noires travaillant comme domestiques, Acciari (2020) présente que « les femmes interviewées partagent toutes le sentiment d’être invisibles, dévalorisées et non reconnues pour le travail qu’elles effectuent » (p. 127)

#### Relation à soi et aux autres

Une dernière caractéristique essentielle à la reconnaissance de l’intersectionnalité au travail concerne la relation à soi et aux autres. Celle-ci met en lumière la manière dont les identités sont contextuellement exprimées ou modifiées en fonction des dynamiques sociales et des interactions quotidiennes en milieu de travail. Contrairement à l’expérience subjective idiosyncratique, qui se centre sur la perception interne de la personne, ou aux relations de pouvoir, qui soulignent les structures hiérarchiques, cette dimension porte sur l’adaptation identitaire selon les personnes côtoyées, les groupes en présence et les perceptions mutuelles. Les inégalités intersectionnelles se reproduisent à travers les interactions entre collègues, gestionnaires et autres acteurs du milieu de travail (Acker, 2011; Alberti & Iannuzzi, 2020). Luiz et Terziev (2024) illustrent que certaines personnes ajustent la visibilité de leurs identités selon leur environnement social immédiat. Par exemple, un participant à leur étude mentionne que « les identités sont souvent contextuelles ; mon orientation sexuelle peut devenir plus importante dans un groupe d’hommes cisgenres, ma “blancheur” devient plus importante dans un groupe d’Africains majoritairement noirs » (p.306).

Cette relation identitaire est influencée par la perception de soi, mais aussi par la manière dont on croit être perçu par les autres (Acker, 2011; Luiz & Terziev, 2024). Cette tension entre perception personnelle et reconnaissance sociale peut générer des ajustements stratégiques ou des conflits identitaires dans les interactions. Dans certains contextes, ces dynamiques relationnelles affectent les trajectoires professionnelles. Par exemple, dans le domaine sportif, Borland et Bruening (2010) soulignent que les femmes noires sont fréquemment perçues comme étant plus aptes à assumer des rôles d’athlètes plutôt que d’entraîneuses. Ces perceptions influencent à la fois les opportunités réelles d’emploi et la façon dont ces femmes perçoivent leurs propres compétences et aspirations professionnelles.

Enfin, cette relation à soi et aux autres peut générer des conflits internes lorsqu’il y a un décalage entre les identités vécues dans le cadre professionnel et celles auxquelles les individus s’identifient dans d’autres sphères de leur vie. Comme l’explique Summerville (2022):

« […] les employés vivent différemment leur identité dans les structures organisationnelles, où ils doivent se débattre avec la nature imbriquée des significations socialement construites associées à leur race, leur sexe et leur identité professionnelle, et comment ces significations peuvent entrer en conflit avec les définitions personnelles auxquelles ils s’attribuent en dehors du cadre du travail » (p.94).

Ainsi, la relation à soi et aux autres est une dimension centrale de l’intersectionnalité au travail : elle montre comment les identités se vivent, se négocient ou s’ajustent dans les relations sociales quotidiennes, révélant des dynamiques complexes d’appartenance, d’exclusion ou de reconnaissance, souvent invisibles mais profondément structurantes.

En somme, l’opérationnalisation du concept d’intersectionnalité au travail repose sur cinq caractéristiques interreliées, qui, bien que distinctes, se renforcent mutuellement pour offrir une compréhension riche et nuancée des dynamiques professionnelles. D’abord, l’interrelation des identités multiples permet de saisir comment les divers éléments identitaires (p. ex., genre, ethnicité, handicap) ne s’expriment pas isolément, mais s’entrecroisent pour moduler l’expérience de chacun. Cette perspective est intimement liée à la dynamique entre privilèges et désavantages, qui révèle comment les positions sociales confèrent des avantages à certains tout en en défavorisant d’autres, parfois simultanément, chez une même personne. Ces effets se manifestent au sein de rapports de pouvoir, une caractéristique essentielle qui expose la façon dont certaines identités sont valorisées, tandis que d’autres sont systématiquement subordonnées dans les processus décisionnels organisationnels. Parallèlement, l’expérience subjective idiosyncratique souligne l’importance de considérer la singularité du vécu de chaque individu, façonné par la combinaison unique de ses identités et par son contexte de travail. Enfin, la relation à soi et aux autres met en lumière les dimensions interactionnelles et réflexives de l’intersectionnalité, en montrant comment les perceptions mutuelles influencent l’expression identitaire et renforcent ou atténuent les inégalités perçues. Ensemble, ces cinq caractéristiques forment un cadre analytique cohérent et complémentaire, qui permet de reconnaître, comprendre et agir sur les multiples formes d’inégalités vécues dans les milieux de travail contemporains.

#### Contexte sociétal et historique

Plusieurs textes insistent sur l’importance de comprendre les dynamiques identitaires et les expériences d’oppression à travers une lecture ancrée dans l’histoire sociale. L’intersectionnalité ne se résume pas à des expériences contemporaines, mais révèle des mécanismes de domination souvent anciens, perpétués par des structures institutionnelles et normatives. Nos résultats soulignent ainsi que les influences historiques, sociales, politiques, économiques, culturelles et organisationnelles pourraient jouer un rôle dans la façon dont l’intersectionnalité est examinée, définie et vécue. En effet, les définitions de la race, de la classe ou du genre ne sont pas figées ; elles sont plutôt « des catégories abstraites, qui peuvent avoir un contenu différent selon les situations historiques, sociales et politiques » (Acker, 2011, p.6). Cela renvoie à l’origine même du concept d’intersectionnalité, formulé par Crenshaw (1989) dans un contexte juridique nord-américain pour mettre en lumière les formes d’oppressions structurelles vécues par les femmes noires dans le monde du travail. Depuis, le concept s’est élargi et adapté à d’autres contextes, révélant que les formes d’oppression sont toujours inscrites dans des structures sociales et économiques spécifiques.

Par exemple, Acciari (2021), dans une étude sur le travail domestique au Brésil, soulève que ce travail « s’inscrit dans une division historique du travail en fonction du sexe et de la race, qui permet à certains d'accéder à de bons emplois sûrs tout en externalisant le fardeau de la reproduction sociale vers les autres précaires » (p.70). Cette division n’est pas simplement contemporaine, elle est l’héritage de « l'économie (post)coloniale et capitaliste qui a fait des travailleuses et travailleurs domestiques une sous-classe de serviteurs, exclus de la législation du travail depuis des décennies » (Acciari, 2021, p.69). Cela souligne l’existence d’une structure historique et sociale dans laquelle le travail domestique est dévalorisé et délégué aux personnes plus vulnérables, ce qui permet aux individus en bénéficiant de se concentrer sur des carrières plus valorisées par la société. Ces constats soulignent que les structures d’oppression sont enracinées dans des rapports de pouvoir historiques et qu’elles se manifestent encore dans les dynamiques professionnelles actuelles. Ainsi, une analyse intersectionnelle du travail requiert de tenir compte de ces structures et de leur évolution dans le temps, puisqu’elles façonnent les opportunités, les contraintes et les formes de reconnaissance ou d’exclusion que vivent les personnes dans les milieux de travail. Cette perspective historique permet de contextualiser les interrelations identitaires et de mieux comprendre pourquoi certains groupes restent structurellement marginalisés dans les milieux de travail.

## Discussion

La présente étude visait à identifier les caractéristiques opérationnelles du concept de l’intersectionnalité au travail. Cette conceptualisation propose une base théorique permettant aux ergothérapeutes de mieux reconnaître les dynamiques d’oppression qui influencent la participation occupationnelle des personnes dans des contextes professionnels diversifiés. Deux contributions principales émergent de cette étude : (1) la reconnaissance des dynamiques relationnelles invisibles dans les milieux de travail, et (2) l’importance du contexte dans l’analyse des inégalités vécues au travail. Ces contributions peuvent guider à la fois les pratiques des ergothérapeutes pour favoriser une participation au travail juste, équitable et inclusive des personnes, mais aussi la réalisation de nouvelles études en ergothérapie qui portent sur des populations marginalisées.

### Reconnaître les dynamiques relationnelles (in)visibles dans les milieux de travail

Cette contribution vise à reconnaître les dynamiques relationnelles (in)visibles dans les milieux de travail, non pas comme des phénomènes isolés, mais comme les manifestations actuelles de systèmes de domination historiquement enracinés (p. ex., colonialisme, patriarcat, racisme systémique). Ces dynamiques, bien qu’elles puissent paraître subtiles ou implicites, prennent appui sur des structures sociales qui attribuent différemment la valeur, la visibilité et la légitimité à certaines identités dans les contextes professionnels. Ces dynamiques peuvent s’exprimer dans les interactions entre l’individu et le milieu de travail, entre collègues, ou encore dans la manière dont une personne se perçoit et ajuste son comportement. Lorsqu’il est question d’« invisibilité », il ne s’agit pas d’une absence de relations, mais plutôt de mécanismes implicites et difficiles à percevoir directement, comme les biais implicites, les stéréotypes intériorisés ou les normes organisationnelles tacites. Ces éléments, bien qu’ils ne soient pas toujours manifestes ou formellement codifiés, influencent fortement l’expérience vécue par les personnes dans leur milieu professionnel (Atewologun et al., 2016; De los Reyes, 2017).

Les personnes présentant plusieurs identités minoritaires développent souvent des stratégies de gestion identitaire afin de naviguer au sein de ces dynamiques sociales complexes (Nicole & Drolet, 2023). Comme le montrent Atewologun et collaborateurs (2016), cela peut impliquer d’adapter son comportement, par exemple en étant plus assertif pour être entendu, tout en modérant ses réactions afin d’éviter d’être perçu comme agressif, selon les stéréotypes associés à ses identités. Ces ajustements, bien qu’invisibles pour les observateurs externes, témoignent d’un travail identitaire constant, façonné par les rapports de pouvoir et de perception qui traversent les milieux de travail.

L’invisibilité concerne également les expériences intériorisées des personnes stigmatisées, qui peuvent anticiper ou craindre l’exclusion, le rejet ou la discrimination. Remedios et Snyder (2018) décrivent ainsi comment ces perceptions renforcent l’internalisation des normes dominantes, contribuant à des formes d’invisibilité sociale et psychologique. Ces dynamiques difficilement observables sont réelles et structurellement ancrées.

Ces constats soulignent la nécessité, pour les ergothérapeutes, de développer une vigilance accrue envers les signes indirects d’exclusion, tels que l’autocensure, le retrait relationnel, l’hypervigilance identitaire ou les ajustements comportementaux stratégiques. Il s’agit d’être attentif non seulement aux interactions explicites, mais aussi aux silences, aux absences de reconnaissance et aux adaptations invisibles que les personnes doivent opérer pour se conformer à un milieu de travail normatif. La profession reconnaît d’ailleurs l’importance de prendre en compte les histoires, les cultures, les identités et les normes sociales dans l’analyse des occupations (Egan & Restall, 2022). Une lecture intersectionnelle, en intégrant ces dimensions, permet de mieux comprendre comment des injustices occupationnelles émergent de rapports sociaux implicites, mais profondément enracinés dans l’organisation du travail (Pooley & Beagan, 2021; Smith et al., 2025). En ce sens, l’analyse intersectionnelle s’inscrit pleinement dans l’approche ergothérapique, en outillant les ergothérapeutes à reconnaître la complexité des expériences vécues et à adapter leurs stratégies d’intervention (Gerlach, 2015).

Les caractéristiques opérationnelles de l’intersectionnalité identifiées dans cette étude offrent des leviers concrets pour soutenir la pratique des ergothérapeutes. Cette reconnaissance des dynamiques relationnelles (in)visibles peut notamment se traduire par : (1) l’adaptation des outils d’évaluation pour inclure des questions sur les expériences d’oppression, d’appartenance ou de reconnaissance en contexte de travail ; (2) l’allocation d’un temps suffisant en entretien pour explorer les trajectoires sociales et professionnelles, au-delà des seules limitations fonctionnelles ; (3) la mise en place de pratiques de co-analyse avec les personnes accompagnées, afin de mieux comprendre leurs réactions comme des réponses légitimes à des environnements oppressifs, plutôt que comme des signes de dysfonctionnement individuel ; et (4) le développement de compétences réflexives chez les ergothérapeutes en matière de pouvoir, de privilège et de normativité, notamment par des espaces structurés de réflexion partagée, comme les communautés de pratique.

Cet ajustement de la pratique s’inscrit dans un mouvement plus large visant à promouvoir la justice, l’équité et les droits des personnes (Restall, 2024). L’approche intersectionnelle permet ainsi de dépasser les modèles centrés uniquement sur les limitations individuelles ou les caractéristiques de l’environnement physique, en intégrant les rapports de pouvoir, les normes sociales implicites et les obstacles structurels à la participation occupationnelle (Yao et al., 2022). Cette compréhension enrichit également le rôle de plaidoyer des ergothérapeutes auprès des milieux de travail, des gestionnaires et des instances politiques. Elle les aide à identifier les mécanismes structurels de discrimination, souvent banalisés ou méconnus, et à promouvoir des environnements inclusifs, équitables et sensibles à la diversité. En intégrant cette posture critique, les ergothérapeutes peuvent jouer un rôle actif dans la transformation des systèmes qui entravent la participation des personnes (Angell, 2014; Farias et al., 2016), notamment dans un contexte de recul des politiques d’équité, de diversité et d’inclusion. Les ergothérapeutes peuvent alors nommer les effets de ce recul sur les personnes marginalisées, soutenir des revendications collectives et proposer des actions de sensibilisation sur les dynamiques d’exclusion intersectionnelle au sein des organisations.

### L’importance du contexte dans l’analyse des inégalités vécues au travail

Les résultats de notre étude confirment l’importance centrale de différentes dimensions du contexte dans la compréhension et la mobilisation du concept d’intersectionnalité au travail. Ces dimensions ancrent l’intersectionnalité dans une réalité située, en cohérence avec l’idée que les identités et les formes d’oppression qui en découlent ne peuvent être dissociées de l’espace-temps dans lequel elles s’expriment (Acker, 2011; Chilakala et al., 2022; Smith, 2024). Plus précisément, notre étude met en valeur l’importance spécifique du contexte organisationnel, en tant qu’espace de socialisation professionnelle et d'expression des normes, des valeurs et des rapports de pouvoir. Ce contexte aurait une influence prépondérante sur l’expérience d’appartenance à des diversités dans le milieu de travail. Par exemple, une culture organisationnelle inclusive peut favoriser l’expression des diversités intersectionnelles, augmenter le sentiment de bien-être de la main-d’œuvre et permettre aux milieux de travail de tirer des bénéfices des diversités (Downie, 2010). À l’inverse, une culture injuste, non équitable et non inclusive peut entrainer les individus à camoufler des caractéristiques de leurs diversités, ainsi qu’amplifier la discrimination et les effets négatifs s’y rattachant (Byrd, 2018). La prise en compte des dimensions environnementales et historiques du contexte est d’autant plus importante en ergothérapie, discipline centrée sur la participation occupationnelle dans un contexte donné. Elle permet aux ergothérapeutes de reconnaître que les inégalités vécues ne sont pas seulement individuelles, mais le fruit de systèmes d’oppression construits et reproduits historiquement (Nixon, 2019). En intégrant cette perspective, les interventions peuvent être mieux adaptées aux réalités vécues par les personnes ayant des identités intersectionnelles, et viser une transformation sociale orientée vers plus d’équité et de justice.

Cette sensibilité au contexte se retrouve dans plusieurs travaux menés par des ergothérapeutes qui ont exploré des concepts émergents ou complexes tels que la prévention intégrée au travail (Lecours et al., 2023), l’estime de soi au travail (Pierce & Gardner, 2004), ou encore l’identité occupationnelle (Hansson et al., 2022). Bien qu’encore peu nombreux, les ergothérapeutes qui mènent des travaux théoriques pour définir des concepts émergents ou encore pour clarifier des concepts vagues ou mal compris offrent une contribution notable à l’avancement des connaissances par l’intérêt qu’ils portent au contexte. Puisque les concepts se doivent d’être définis en regard des contextes dans lesquels ils sont mobilisés (Walker & Avant, 2019), nous encourageons les ergothérapeutes à mettre à profit leurs compétences et leurs expertises pour rehausser les connaissances sur les concepts.

### Forces, limites et perspectives de recherche

Cette étude constitue une contribution originale à l'avancement des connaissances sur l’intersectionnalité dans le contexte du travail, en proposant une conceptualisation fondée sur des caractéristiques opérationnelles. L'une des forces majeures de cette étude réside dans sa capacité à traduire un concept théorique complexe en repères concrets, utiles pour les ergothérapeutes souhaitant mieux comprendre les inégalités vécues dans les milieux de travail. L’utilisation d’un examen de la portée rigoureux, incluant des documents de sources variées (p. ex., articles scientifiques, site web) issus des disciplines de la santé, de la sociologie, de la psychologie et de l’administration, a permis de brosser un portrait large et nuancé du concept dans le contexte du travail. Enfin, un texte en français sur un sujet issu majoritairement de la littérature anglophone est une force permettant de rendre accessible les informations sur un concept important à la communauté ergothérapique francophone.

Cependant, comme le soulignent Collins (2015) et d'autres auteurs critiques, les personnes qui mènent des travaux de recherche ne peuvent ignorer l’influence de leur posture, de leurs expériences personnelles et du contexte sociopolitique sur leurs travaux. Tel que mentionné dans la section Méthodologie, notre équipe est relativement homogène sur le plan ethnoracial et éducatif, ce qui peut limiter notre sensibilité à certaines réalités vécues par des groupes marginalisés. Cette posture peut créer des angles morts dans notre analyse. En revanche, elle nous a aussi permis de réfléchir à un autre aspect de l’intersectionnalité, moins souvent abordé : celui du privilège, et de la manière dont il s’exprime dans les milieux de travail comme dans le monde de la recherche. Cette prise de conscience a alimenté, tout au long de la recherche, une réflexion continue sur la manière dont les rapports de pouvoir influencent non seulement notre objet d’étude, mais aussi notre façon de faire de la recherche. Elle souligne, plus largement, la nécessité de former les ergothérapeutes à reconnaître leur position dans les systèmes de pouvoir et à analyser les dimensions structurelles qui façonnent leurs relations de travail. Cela passe notamment par des approches pédagogiques critiques, des outils réflexifs et des espaces de dialogue qui permettent de situer leur pratique au sein d’enjeux sociaux plus larges. En ce sens, cette recherche constitue non seulement un exercice analytique, mais aussi un engagement envers une démarche transformatrice.

Certaines limites méthodologiques doivent aussi être reconnues. Une large proportion des documents retenus provient d’Amérique du Nord, principalement des États-Unis. Cette concentration géographique reflète l’origine du concept, mais elle limite la transférabilité des résultats à des contextes culturels, organisationnels ou juridiques différents. Aussi, les données de cette étude constituent des documents publiés, ce qui fait que nous sommes contraints à travailler avec les informations disponibles. Ce faisant, certaines idées peuvent manquer de détails par non-disponibilité. Enfin, la majorité des études examinées abordent l’intersection entre le sexe et l’ethnicité, laissant en marge d’autres formes de diversité (p. ex., orientation sexuelle, neurodivergence) ou la combinaison de plus de deux identités minoritaires. Cette limite ouvre des avenues de recherche futures portant sur l’intersectionnalité plurielle ou multidimensionnelle. Bien que la majorité des écrits recensés proviennent d'autres disciplines, les résultats de cette étude offrent un potentiel d'intégration prometteur dans le champ de l'ergothérapie. En cartographiant des savoirs issus de champs variés, comme la sociologie, la santé publique ou l’éducation, cet article permet de dégager des connaissances transférables et de faire émerger des angles morts utiles à la pratique ergothérapique. Les caractéristiques opérationnelles identifiées peuvent ainsi soutenir les ergothérapeutes dans l’analyse des situations intersectionnelles vécues par les personnes en contexte de travail. Elles fournissent des repères concrets pour mieux comprendre les dynamiques d’oppression et de privilège, sans toutefois prétendre révolutionner la pratique. Des travaux futurs pourraient explorer plus spécifiquement la transposition de ces éléments dans des contextes propres à notre profession, afin de soutenir de façon plus ciblée les interventions visant à réduire les effets négatifs des situations d’exclusion vécues par les personnes aux identités marginalisées.

## Conclusion

Cette étude a permis d’identifier les caractéristiques opérationnelles de l’intersectionnalité au travail pouvant guider les ergothérapeutes dans l’accompagnement des personnes et des organisations vers une participation au travail juste, équitable et inclusive ainsi que vers la conduite de nouvelles études portant sur des populations marginalisées. En révélant les dynamiques relationnelles, souvent invisibles, et l’influence des contextes historiques, organisationnels et sociaux, l’étude offre un cadre structuré pour analyser les inégalités vécues par les personnes ayant des identités croisées dans les milieux de travail. Pour les ergothérapeutes, cette étude constitue un outil d’analyse et d’intervention, qui permet d’évaluer les obstacles structurels à la participation occupationnelle et d’élaborer des stratégies adaptées à la diversité des expériences.

Cette étude invite la communauté ergothérapique à poursuivre le développement de concepts émergents, en tenant compte des contextes dans lesquels ils prennent sens, et à cultiver une posture réflexive et critique sur les rapports de pouvoir qui traversent les environnements de travail. L’intersectionnalité, bien qu’elle constitue un levier d’analyse puissant, ne porte un véritable potentiel transformateur que si elle est mobilisée dans des conditions structurelles et politiques qui en soutiennent l’impact. Dans un contexte où les principes d’équité, de diversité et d’inclusion sont parfois remis en question ou instrumentalisés, il ne suffit plus de reconnaître les injustices : il faut s’engager activement dans des pratiques collectives et structurelles orientées vers la transformation des milieux, plutôt que vers l’adaptation des individus à un système inéquitable. En intégrant cette posture critique, l’ergothérapie renforce sa capacité à répondre aux besoins des personnes les plus marginalisées et à promouvoir des milieux de travail plus justes pour toutes et tous.

## Messages clés

- Adopter une perspective intersectionnelle aide les ergothérapeutes à reconnaître les sources d’oppression et à proposer des interventions adaptées aux identités croisées.
- Les caractéristiques de l’intersectionnalité au travail ne peuvent être comprises sans considérer les contextes historiques, sociaux et organisationnels.
- L’expertise des ergothérapeutes en analyse des occupations dans leur contexte les positionne comme acteurs-clés dans le développement théorique sur l’équité, la diversité, l’inclusion et la justice au travail.

## Supplemental Material

sj-docx-1-cjo-10.1177_00084174251383837 - Supplemental material for Caractériser l'intersectionnalité au travail: des repères opérationnels pour soutenir les ergothérapeutes Supplemental material, sj-docx-1-cjo-10.1177_00084174251383837 for Caractériser l'intersectionnalité au travail: des repères opérationnels pour soutenir les ergothérapeutes by Alexandra Lecours, Andrée-Anne Drolet, Lily Bellehumeur-Béchamp, Marie-Josée Drolet, Claude Vincent, Samuel Turcotte and Dimitri Léonard in Canadian Journal of Occupational Therapy

## Notes

1. Pour les documents publiés en anglais, les citations intégrées dans la section des résultats ont été traduites en français pour harmoniser avec la langue de rédaction de cet article.

2. N/S = non spécifié
