# Supplementary material for: Characterizing Intersectionality at Work: Operational Guidelines to Support Occupational Therapists
Source: Can J Occup Ther. 2025 Oct 21;93(3):341–57. [Article in French] doi: 10.1177/00084174251383837 (PMC13400804; doi:10.1177/00084174251383837)
Supplement: sj-docx-1-cjo-10.1177_00084174251383837 - Supplemental material for Caractériser l'intersectionnalité au travail: des repères opérationnels pour soutenir les ergothérapeutes [file sj-docx-1-cjo-10.1177_00084174251383837.docx]

**Annexe 1 - Références des manuscrits inclus dans l’examen de la portée**

Acciari, L. (2020). «Le travail domestique est aussi une profession!» La lutte des travailleuses domestiques au Brésil pour l’égalité des droits. *Revue internationale des études du développement, 242*(2), 119-141. <https://doi.org/10.3917/ried.242.0119>.

Acciari, L. (2021). Practicing intersectionality: Brazilian domestic workers’ strategies of building alliances and mobilizing identity. *Latin American Research Review*, *56*(1), 67-81. <https://doi.org/10.25222/larr.594>

Acker, J. (2011). Theorizing gender, race, and class in organizations. Dans Jeanes, E., Knights, D. et Martin, P. Y. (dir.), *Handbook of gender, work and organization* (p.65-80). Wiley.

Alberti, G. et Iannuzzi, F. E. (2020). Embodied intersectionality and the intersectional management of hotel labour: The everyday experiences of social differentiation in customer‐oriented work. *Gender, Work & Organization*, *27*(6), 1165-1180. <https://doi.org/10.1111/gwao.12454>

Alt, N. P., Wax, A., Brush, O. T. et Magalona, J. (2024). Asian American women’s workplace experiences: A review and application of gendered race theory and the intersectional prototypicality model. *American Behavioral Scientist*, *0*(0). <https://doi-org.biblioproxy.uqtr.ca/10.1177/00027642241231313>

Riza Arifeen, S. et Gatrell, C. (2013). A blind spot in organization studies: Gender with ethnicity, nationality and religion. *Gender in Management: An International Journal*, *28*(3), 151-170. <https://doi.org/10.1108/GM-01-2013-0008>

Borland, J. F. et Bruening, J. E. (2010). Navigating barriers: A qualitative examination of the under-representation of Black females as head coaches in collegiate basketball. *Sport Management Review*, *13*(4), 407-420. <https://doi.org/10.1016/j.smr.2010.05.002>

Browne, I. et Misra, J. (2003). The intersection of gender and race in the labor market. *Annual review of sociology*, *29*(1), 487-513. <https://doi.org/10.1146/annurev.soc.29.010202.100016>

Callico, C. (2020). *L'intersectionnalité, du concept au concret*. Centre d'Action Laique. <https://www.laicite.be/magazine-article/lintersectionnalite-concept-concret/>

Chilakala, A., Camacho-Rivera, M. et Frye, V. (2022). Experiences of race-and gender-based discrimination among Black female physicians. *Journal of the National Medical Association*, *114*(1), 104-113. <https://doi.org/10.1016/j.jnma.2021.12.008>

Corlett, S. et Mavin, S. (2014). Intersectionality, identity and identity work: Shared tenets and future research agendas for gender and identity studies. *Gender in Management: An International Journal*, *29*(5), 258-276.  <https://doi.org/10.1108/GM-12-2013-0138>

De los Reyes, P. (2017). Working life inequalities: Do we need intersectionality? *Society, Health & Vulnerability*, *8*(sup1). https://doi.org/10.1080/20021518.2017.1332858

Dhanani, L. Y., Totton, R. R., Hall, T. K. et Pham, C. T. (2024). Visible but hidden: An intersectional examination of identity management among sexual minority employees. *Journal of management*, *50*(3), 949-978. [https://doi.org/10.1177/01492063221121787](https://psycnet.apa.org/doi/10.1177/01492063221121787)

DiStasio, C. (s.d.). *Intersectionnalité et raison de son importance pour votre organisation*. ADP Canada. <https://insights.adp.ca/fr/intersectionnalite-et-raison-de-son-importance-pour-votre-organisation/>

Fuentes, K., Hsu, S., Patel, S. et Lindsay, S. (2024). More than just double discrimination: A scoping review of the experiences and impact of ableism and racism in employment. *Disability and Rehabilitation*, *46*(4), 650-671. <https://doi.org/10.1080/09638288.2023.2173315>

Gallot, F., Noûs, C., Pochic, S. et Séhili, D. (2020). L’intersectionnalité au travail. *Travail, genre et sociétés*, *44*(2), 25-30. <https://doi.org/10.3917/tgs.044.0025>

Institute for Gender and the Economy. (2019). *Intersectionnalité et répercussions sur l’équité entre les sexes en milieu de travail*. <https://www.gendereconomy.org/wp-content/uploads/2019/04/GATE_Intersectionnalite%CC%81-et-re%CC%81percussions_Re%CC%81sume%CC%81DeRecherche_Online.pdf>

Kriger, D., Keyser-Verreault, A., Joseph, J. et Peers, D. (2021). *Le Cadre d’opérationnalisation de l’intersectionnalité : Un outil pour les administrateurs sportifs*. Centre de documentation pour le sport (SIRC) <https://sirc.ca/fr/blog/le-cadre-doperationnalisation-de-lintersectionnalite/>

Lavaysse, L. M., Probst, T. M. et Arena Jr, D. F. (2018). Is more always merrier? Intersectionality as an antecedent of job insecurity. *International journal of environmental research and public health*, *15*(11), 2559. <https://doi.org/10.3390/ijerph15112559>

Luiz, J. M. et Terziev, V. (2024). Axes and fluidity of oppression in the workplace: Intersectionality of race, gender, and sexuality. *Organization*, *31*(2), 295-315. <https://doi.org/10.1177/13505084221098252>

McDowell, J. et Carter-Francique, A. (2017). An intersectional analysis of the workplace experiences of African American female athletic directors. *Sex Roles*, *77*(5-6), 393-408. <https://doi.org/10.1007/s11199-016-0730-y>

Mooney, S., Ryan, I. et Harris, C. (2017). The intersections of gender with age and ethnicity in hotel careers: Still the same old privileges? *Gender, Work & Organization*, *24*(4), 360-375. [https://doi.org/10.1111/gwao.12169](https://psycnet.apa.org/doi/10.1111/gwao.12169)

Randstad. (2020). *L'importance de l'intersectionnalité au travail*. <https://www.randstad.ca/fr/employeurs/tendances-employeur/promouvoir-les-femmes/limportance-de-lintersectionnalite-au-travail/>

Rodriguez, J. K., Holvino, E., Fletcher, J. K. et Nkomo, S. M. (2016). The theory and praxis of intersectionality in work and organisations: where do we go from here? *Gender, Work and Organization 23*(3), 201-222. <https://doi.org/10.1111/gwao.12131>

Rosette, A., Ponce de Leon, R., Zhou Koval, C. et Harrison, D. (2019). Intersectionality: Connecting experiences of gender with race at work. *Research in Organizational Behavior*, *38*, 1-22. <https://doi.org/10.1016/j.riob.2018.12.002>

Salter, N. P., Sawyer, K. et Gebhardt, S. T. (2021). How does intersectionality impact work attitudes? The effect of layered group memberships in a field sample. *Journal of Business and Psychology*, *36*(6), 1035-1052. [https://doi.org/10.1007/s10869-020-09718-z](https://psycnet.apa.org/doi/10.1007/s10869-020-09718-z)

Summerville, K. (2022). *The Multidimensional Self at Work: An Intersectional Examination of Identity Conflict and Authenticity among Black and White Men and Women* [thèse de doctorat, University of North Carolina at Charlotte]. ProQuest Dissertations & Theses Global Closed Collection. <file:///C:/Users/HP/Downloads/Summerville_uncc_0694D_13225.pdf>

Van Buren III, H. J. (2015). Intersectionality in the workplace. Dans Syed, J. et Ozbilgin, M. *Managing diversity and inclusion: an international perspective* (p.354-382). SAGE Publications.

Wang, Y. (2015). The mystery revealed—Intersectionality in the black box: An analysis of female migrants' employment opportunities in urban China. *Hypatia*, *30*(4), 862-880. <https://doi.org/10.1111/hypa.12171>
